# Quality Assessment of Therapeutic Drug Monitoring Assays of Therapeutic Antibodies Across Europe: An Update

**DOI:** 10.1111/bcpt.70129

**Published:** 2025-10-06

**Authors:** James Bluett, Merita Rumano, María José Martínez Becerra, Floris Loeff, Mehmet Itik, David Ternant, Céline Desvignes, Denis Mulleman, Silje Skrede

**Affiliations:** ^1^ Versus Arthritis Centre for Genetics and Genomics, Centre for Musculoskeletal Research The University of Manchester, Manchester Academic Health Science Centre Manchester UK; ^2^ Manchester Foundation Trust, Manchester Academic Health Science Centre Manchester UK; ^3^ European Network on Optimising Treatment with Therapeutic Antibodies in Chronic Inflammatory Diseases (ENOTTA); ^4^ Department of Biology, Faculty of Natural Sciences University of Tirana Tirana Albania; ^5^ Department of Basic Sciences and Public Health Department, Faculty of Medicine University of Medicine Tirana Albania; ^6^ Immunology Laboratory University Hospital Fundación Jiménez Díaz Madrid Spain; ^7^ Sanquin Diagnostic Services Amsterdam Netherlands; ^8^ Deparment of Aerospace Engineering Dokuz Eylul University Izmir Turkey; ^9^ CHRU de Tours Plateforme Recherche Centre Pilote de Suivi Biologique des Traitements par Anticorps (CePiBAc) Tours France; ^10^ CHRU de Tours Service de Pharmacologie Médicale Tours France; ^11^ Inserm U1327 ISCHEMIA ‘Membrane Signalling and Inflammation in Reperfusion Injuries’ Université de Tours Tours France; ^12^ Center for Molecular Biophysics, UPR CNRS 4301, Nanomedicines and Nanoprobes Department University of Tours Tours France; ^13^ Centre Hospitalier Régional Universitaire de Tours Service de Rhumatologie Tours France; ^14^ Department of Clinical Science, Faculty of Medicine University of Bergen Bergen Norway; ^15^ Department of Medical Biochemistry and Pharmacology Haukeland University Hospital Bergen Norway

**Keywords:** biological DMARD, disease‐modifying drugs (DMARDs), immune‐mediated inflammatory diseases (IMIDs), therapeutic drug monitoring (TDM)

## Abstract

Immune mediated inflammatory diseases (IMIDs) are common, chronic, inflammatory diseases. There has been an expansion of monoclonal antibodies to treat the disease. However, the response is not universal; reasons for non‐response may include suboptimal drug concentrations for which therapeutic drug monitoring (TDM) may lead to improved outcomes. Several laboratory assays are available to measure biologic drug concentrations, but variation in assay accuracy may introduce bias. External quality assessments (EQA) provide assurance of the performance of a laboratory test. The aim of this work was to survey current quality assessment procedures that are in place in laboratories undertaking TDM of therapeutic antibodies for the treatment of IMIDs to guide clinical decision making across Europe. A survey was sent out to institutions undertaking TDM across Europe. In total, 26 institutions responded; the vast majority (96.2%) of institutions utilize an internal quality control, with 42% of institutions not reporting participation in a national EQA scheme. Barriers to EQA participation included insufficient information about relevant organizations and financial constraints. These results demonstrate that, although TDM assay performance is well controlled locally, better access to EQA and standards may further aid the translatability of results between laboratories and aid the adoption of published reference values.

## 1 Introduction

Immune mediated inflammatory diseases (IMIDs) affect 5%–7% of the western world [1]. A greater understanding of the immuno‐pathogenesis of IMIDs has led to more targeted therapies. Monoclonal antibodies are widely used to treat IMIDs across various medical disciplines, including gastroenterology, rheumatology, dermatology, neurology, and paediatrics. However, the response is not universal; there are a number of possible reasons for non‐response, one of which includes variation in drug exposure. Most of the advanced therapies have standardized dosing; therapeutic drug monitoring (TDM) may lead to improved outcomes for patients so that the patient receives the right dose at the right time. Several laboratory techniques and assays are available to measure biologic drug levels, but there is no gold standard assay, and only for a few drugs are reference standards available [2]. Variation in the accuracy of the assays may introduce bias in the dosage adjustment and be a significant barrier to accurate TDM.

In 2022, the European Alliance of Associations for Rheumatology (EULAR) published points‐to‐consider for TDM of biologics in inflammatory rheumatic and musculoskeletal diseases [3] recommending that laboratories performing the assays should be ‘familiar with the test characteristics’ and that assays must ‘be validated in accordance to (inter)national standards of practice’. External quality assessments (EQA) are evaluations comparing a laboratory test result to a second site and provide assurance of the performance of the laboratory test [4]. It is not yet known, however, the degree of variation of assays used across Europe and quality assessments that are in use in routine practice.

European Network on Optimizing Treatment with Therapeutic Antibodies in chronic inflammatory diseases (ENOTTA) network is a European Cooperation in Science and Technology (COST) Action with the aim to create an interdisciplinary, pan‐European Network to defragment and structure the scientific research in TDM established in 2022 [5]. Currently, 35 COST countries are included in the network. A recent survey performed by ENOTTA received responses from 63 unique respondents across 24 countries and highlighted the current landscape of biologic analytics, revealing active laboratories in 19 countries. The survey results indicated that the most commonly analysed biologics were infliximab and adalimumab, although a wide range of other biologics were also included. Laboratories employed a variety of analytical methods, with a majority using commercial assays, while others utilized in‐house assays or a combination of both [6]. An overview of active laboratories, although by no means exhaustive, can be found here: https://enotta.eu/centers‐with‐tdm‐facilities/.

The objective of this follow‐up project was to survey current quality assessment procedures that are in place in laboratories undertaking TDM of therapeutic antibodies for the treatment of IMIDs to guide clinical decision making across Europe.

## 2 Methods

Institutions undertaking TDM of biologics for IMID indications were invited to participate. Institutions were identified from the list of respondents to a previous survey [6] and through professional networks. TDM institutions were surveyed from June to November 2024. Data collected included

- Current assays in use for therapeutic antibodies
- Presence of internal quality control
- Participation in national or international EQA scheme and for which therapeutic antibodies
- Barriers to participate more actively in EQA schemes

The survey was designed by researchers with expertise in assay development (J.B. and S.S.). Data were collected by Google Forms and manually curated by the authors; the full list of questions is presented in the supplementary. The study was conducted in accordance with the Basic & Clinical Pharmacology & Toxicology policy for experimental and clinical studies [7].

## 3 Results

**FIGURE 1:**
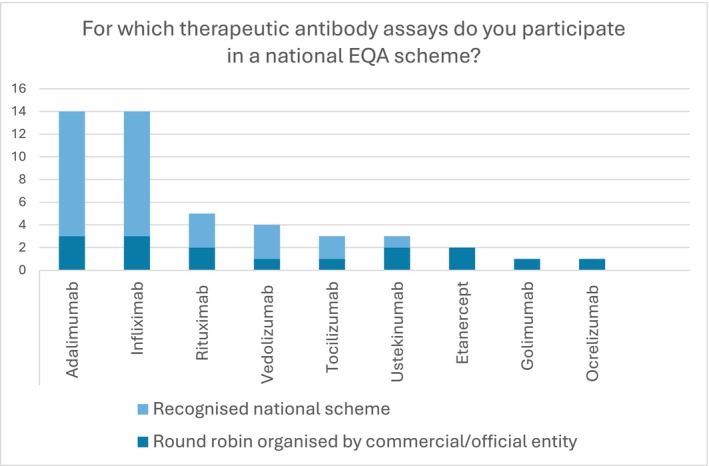
Survey responses of therapeutic antibody assays that laboratories participate in a national EQA scheme.

In total, laboratory employees from 26 institutions responded. The following countries were represented: Croatia (*n* = 1), France (*n* = 7), Spain (*n* = 4), Serbia (*n* = 4), Sweden (*n* = 1), United Kingdom (*n* = 1) and missing (*n* = 8). The vast majority of institutions utilize an internal quality control (96.2%, *n* = 25). Among the assays employed, Enzyme Linked Immunosorbent Assay (ELISA) (69.2% *n* = 18) was the most common, with other laboratories reporting the use of immunofluorometric assays (*n* = 1), chemiluminescence (*n* = 3), cytometry (*n* = 1), LC–MS/MS (*n* = 1) or a combination of assays (*n* = 2). 42% of institutions (*n* = 10; unknown = 1) did not report participation in a national EQA scheme. Among the institutions participating in a national EQA scheme, adalimumab and infliximab were the most commonly measured therapeutic antibodies using recognized national protocols (Figure 1). The frequency of EQA participation varied from once to eight times per year and the number of levels per antibody ranged from one to three.

**FIGURE 2:**
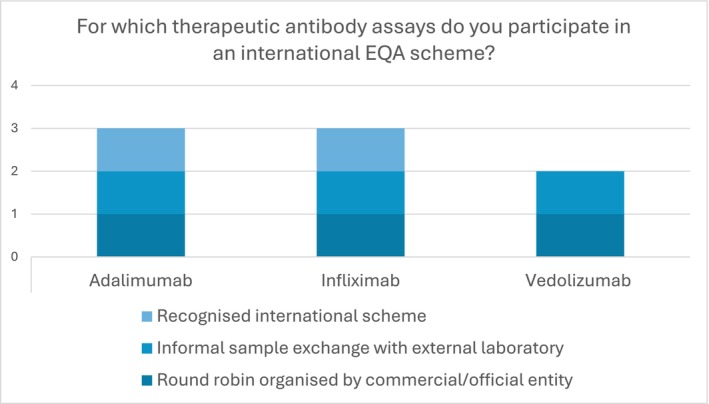
Survey responses of therapeutic antibody assays that laboratories participate in an international EQA scheme.

Four institutions responded that they participated in an international EQA scheme, and again, the majority of therapeutic antibodies using this scheme were adalimumab and infliximab (Figure 2). The number of levels per antibody ranged from one to two. Combined, 31% (*n* = 8) laboratories did not report participation in a national or international EQA scheme.

Among respondents, barriers to EQA participation included insufficient information about relevant organizations (38%; *n* = 10), financial constraints (38%; *n* = 10), time limitations (8%; *n* = 2), absence of networks for informal sample exchange (8%; *n* = 2) and limited experience with EQA (4%; *n* = 1).

## 4 Discussion

Analyses of serum concentrations of biologic drugs as part of TDM in the follow‐up of patients treated for chronic inflammatory diseases are offered by a number of laboratories across Europe. The diversity of analytical methods employed, ranging from commercial assays to in‐house techniques, together with the lack of reference materials, poses a risk for inter‐laboratory variability in TDM. Analytical variability can introduce bias in dosage adjustments, thereby posing a barrier to the accuracy of TDM. The vast majority of laboratories reported internal quality control measures were in place. Whilst internal quality control helps to monitor consistency in the measurement within an individual laboratory, External Quality Assessment schemes provide independent assurance to laboratories that the assay is accurate and that the laboratory test is being performed adequately, and may be considered particularly important when a variety of methods and assays are utilized. The results of our survey demonstrate a wide variability in assays employed in TDM and that approximately half of respondents participated in a national EQA, with far fewer participating at the international level. It is unlikely that one assay will emerge as the gold standard in the near future, and therefore it is vital that laboratories are aware of the individual assay limitations and the potential benefits of EQA schemes to provide assurances of accuracy. Barriers to EQA participation were identified that included lack of information concerning relevant organizations, which may represent lack of national EQA schemes in the countries represented and cost.

Several limitations deserve mentioning. ENOTTA conducted a survey on TDM assays 1 year prior to the current survey, which had a significantly higher number of respondents. Participants may have a higher threshold for responding to a second survey. Moreover, the topic of the present survey may introduce inherent bias, as those not participating in EQA schemes might refrain from responding. To mitigate the latter, respondents were allowed to remain anonymous. Indeed, we received a number of responses from laboratories that do not participate in EQA schemes.

In conclusion, our international European survey of TDM institutions has shown that TDM assays often lack EQA assurances, which may affect TDM results when clinicians are making treatment decisions and be a barrier to international efforts to establish clinical trials evaluating the effectiveness of TDM‐guided interventions. ENOTTA aims to aid laboratories in overcoming barriers to EQA participation by creating networks and increasing awareness of this issue through physical and digital meetings, webinars and assessment of present methodologies and practices.

## Conflicts of Interest

James Bluett: reports a research grant award from Pfizer (awarded 2018) and in the last 3 years travel/conference fees from UCB, Fresenius Kabi, and Novartis. Merita Rumano: None declared. María José Martínez Becerra: None declared. Floris Loeff: As per my current affiliation. Sanquin Diagnostic Services provides biologics TDM service testing for EU hospitals; Sanquin Diagnostic Services provides bioanalytical CRO services to pharmaceutical companies. No personal grants were received. Mehmet Itik: None declared. David Ternant: David Ternant has given lectures on behalf of his institution to Novartis, Sanofi, Lundbeck, and Amgen outside of the submitted work. Céline Desvignes: None declared. Silje Skrede: None declared. Denis Mulleman: reports travel/conference fees from Celltrion healthcare to attend the French Society for Rheumatology 2022 annual meeting in the last 3 years.

## Supporting information


**Data S1:** Supporting Information.

## Data Availability

Research data are not shared.
