# Supplementary material for: Quality Assessment of Therapeutic Drug Monitoring Assays of Therapeutic Antibodies Across Europe: An Update
Source: Basic Clin Pharmacol Toxicol. 2025 Oct 6;137(5):e70129. doi: 10.1111/bcpt.70129 (PMC12498310; doi:10.1111/bcpt.70129)
Supplement: Supplementary file 1 — Data S1: Supporting Information. [file BCPT-137-0-s001.docx]

# COST ENOTTA survey: assays for therapeutic antibodies - External Quality Assessment (EQA)

## Methods and quality controls

Method/assays - which methods are used for therapeutic antibodies and/or anti-drug antibodies in the laboratory? (multiple choice)

- ELISA
- LC-MS/MS
- Immunofluorometric assays
- Nephelometry
- HMSA
- Reporter gene assay
- Lateral flow assay
- Other (free-text box)

Do you have an internal quality control? (multiple choice)

- Yes
- No

Do you participate in EQA or similar arrangement at the national level? (multiple choice)

- Yes
- No

Do you participate in EQA or similar arrangement at the international level? (multiple choice)

- Yes
- No

For which therapeutic antibody assays do you participate in a **national**EQA scheme? (multiple choice)

|  | Round Robin organized by commercial/official entity | Informal sample exchange with external laboratory | Recognized national scheme | Measured but no national EQA scheme in place | Not measured |
| --- | --- | --- | --- | --- | --- |
| Adalimumab (TNF) |  |  |  |  |  |
| Certolizumab (TNF) |  |  |  |  |  |
| Etanercept (TNF) |  |  |  |  |  |
| Golimumab (TNF) |  |  |  |  |  |
| Infliximab (TNF) |  |  |  |  |  |
| Bimekizumab (IL-17) |  |  |  |  |  |
| Brodalumab (IL-17r) |  |  |  |  |  |
| Basiliximab (IL-2r) |  |  |  |  |  |
| Canakinumab (IL-1) |  |  |  |  |  |
| Dupilumab (IL-4r) |  |  |  |  |  |
| Guselkumab (IL-23) |  |  |  |  |  |
| Ixekizumab (IL-17) |  |  |  |  |  |
| Risankizumab (IL-23) |  |  |  |  |  |
| Secukinumab (IL-17) |  |  |  |  |  |
| Tildrakizumab (IL-23) |  |  |  |  |  |
| Tocilizumab (IL-6r) |  |  |  |  |  |
| Ustekinumab (IL12/23) |  |  |  |  |  |
| Abatacept (CD80/86) |  |  |  |  |  |
| Belimumab (BLyS) |  |  |  |  |  |
| Ocrelizumab (CD20) |  |  |  |  |  |
| Rituximab (CD20) |  |  |  |  |  |
| Natalizumab (a4b1) |  |  |  |  |  |
| Vedolizumab (a4b7) |  |  |  |  |  |

How often do you participate per year (rough estimate across analytes)? (free text)

How many levels do you have per antibody? E.g. external control at low-medium-high level. (free text)

Do you know how many laboratories participate? (free text)

How are the results analysed (Z-score, bias, clinical case...)? (free text)

Is there a final report written/sent? (multiple choice)

- Yes
- No
- Other (free text)

What is the name of your Inter-laboratory comparison organization(s)? (free text)

Can you provide the website address? Not mandatory. (free text)

## Specification of EQA approach/scheme - international level

For which therapeutic antibody assays do you participate in an **international**EQA scheme? e.g. NEQAS

|  | Round Robin organized by commercial/official entity | Informal sample exchange with external laboratory | Recognized international scheme | Measured but no international EQA scheme in place | Not measured |
| --- | --- | --- | --- | --- | --- |
| Adalimumab (TNF) |  |  |  |  |  |
| Certolizumab (TNF) |  |  |  |  |  |
| Etanercept (TNF) |  |  |  |  |  |
| Golimumab (TNF) |  |  |  |  |  |
| Infliximab (TNF) |  |  |  |  |  |
| Bimekizumab (IL-17) |  |  |  |  |  |
| Brodalumab (IL-17r) |  |  |  |  |  |
| Basiliximab (IL-2r) |  |  |  |  |  |
| Canakinumab (IL-1) |  |  |  |  |  |
| Dupilumab (IL-4r) |  |  |  |  |  |
| Guselkumab (IL-23) |  |  |  |  |  |
| Ixekizumab (IL-17) |  |  |  |  |  |
| Risankizumab (IL-23) |  |  |  |  |  |
| Secukinumab (IL-17) |  |  |  |  |  |
| Tildrakizumab (IL-23) |  |  |  |  |  |
| Tocilizumab (IL-6r) |  |  |  |  |  |
| Ustekinumab (IL12/23) |  |  |  |  |  |
| Abatacept (CD80/86) |  |  |  |  |  |
| Belimumab (BLyS) |  |  |  |  |  |
| Ocrelizumab (CD20) |  |  |  |  |  |
| Rituximab (CD20) |  |  |  |  |  |
| Natalizumab (a4b1) |  |  |  |  |  |
| Vedolizumab (a4b7) |  |  |  |  |  |

How often do you participate per year (rough estimate across analytes)? (free text)

How many levels do you have per antibody? E.g. external control at low-medium-high level. (free text)

Do you know how many laboratories participate? (free text)

How are the results analysed (Z-score, bias, clinical case...)? (free text)

Is there a final report written/sent? (multiple choice)

- Yes
- No
- Other (free text)

What is the name of your international Inter-laboratory comparison organization(s)? (free text)

Can you provide the website address? Not mandatory. (free text)

## Barriers and contact details

If you would like to participate more actively in EQA schemes, what do you consider the main barriers? (multiple choice)

- Lack of information concerning relevant organs/organizations
- Expenses
- Lack f time
- Lack of support/interest from leader/laboratory manager
- Lack of network for informal sample exchange
- Lack of experience with EQA
- Other (free text)
